# The effect of ultrasound-related stimuli on cell viability in microfluidic channels

**DOI:** 10.1186/1477-3155-11-20

**Published:** 2013-06-28

**Authors:** Dyan N Ankrett, Dario Carugo, Junjun Lei, Peter Glynne-Jones, Paul A Townsend, Xunli Zhang, Martyn Hill

**Affiliations:** 1 Electromechanical Engineering Group, Faculty of Engineering and the Environment, University of Southampton, Southampton SO17 1BJ, UK; 2 Bioengineering Sciences Group, Faculty of Engineering and the Environment, University of Southampton, Southampton SO17 1BJ, UK; 3 Faculty Institute for Cancer Sciences, Faculty of Medical and Health Sciences, Manchester Academic Health Science Centre, University of Manchester, Manchester M13 9WL, UK

**Keywords:** Ultrasound (US), Micro-device, Cardiac myoblasts, Cell viability

## Abstract

**Background:**

In ultrasonic micro-devices, contrast agent micro-bubbles are known to initiate cavitation and streaming local to cells, potentially compromising cell viability. Here we investigate the effects of US alone by omitting contrast agent and monitoring cell viability under moderate-to-extreme ultrasound-related stimuli.

**Results:**

Suspended H9c2 cardiac myoblasts were exposed to ultrasonic fields within a glass micro-capillary and their viability monitored under different US-related stimuli. An optimal injection flow rate of 2.6 mL/h was identified in which, high viability was maintained (~95%) and no mechanical stress towards cells was evident. This flow rate also allowed sufficient exposure of cells to US in order to induce bioeffects (~5 sec), whilst providing economical sample collection and processing times. Although the transducer temperature increased from ambient 23°C to 54°C at the maximum experimental voltage (29 *V*_*pp*_), computational fluid dynamic simulations and controls (absence of US) revealed that the cell medium temperature did not exceed 34°C in the pressure nodal plane. Cells exposed to US amplitudes ranging from 0–29 *V*_*pp*_, at a fixed frequency sweep period (t_sw_ = 0.05 sec), revealed that viability was minimally affected up to ~15 *V*_*pp*_. There was a ~17% reduction in viability at 21 *V*_*pp*_, corresponding to the onset of Rayleigh-like streaming and a ~60% reduction at 29 *V*_*pp*_, corresponding to increased streaming velocity or the potential onset of cavitation. At a fixed amplitude (29 *V*_*pp*_) but with varying frequency sweep period (t_sw_ = 0.02-0.50 sec), cell viability remained relatively constant at t_sw_ ≥ 0.08 sec, whilst viability reduced at t_sw_ < 0.08 sec and minimum viability recorded at t_sw_ = 0.05 sec.

**Conclusion:**

The absence of CA has enabled us to investigate the effect of US alone on cell viability. Moderate-to-extreme US-related stimuli of cells have allowed us to discriminate between stimuli that maintain high viability and stimuli that significantly reduce cell viability. Results from this study may be of potential interest to researchers in the field of US-induced intracellular drug delivery and ultrasonic manipulation of biological cells.

## Background

In ultrasonic cell stimulation micro-devices, the inclusion of ultrasound (US) contrast agent (CA) to enhance US bioeffects or increase cell membrane permeability is common [1]. However, CAs can initiate cavitation and streaming [2] local to cells, potentially compromising cell viability [3,4]. Thus, higher cell viability is likely to be maintained in the absence of CA [5-7]. In our previous study we reported on ultrasonically induced membrane poration of a cardiac myoblast cell line (H9c2) in the absence of CA by generating an ultrasonic field within a biocompatible glass micro-capillary [3]. Notably, high cell viability was maintained in the absence of CA [3]. Following a similar approach, Longsine-Parker *et al.* recently demonstrated effective cell membrane poration in a microfluidic device by combining the action of electric fields and US waves in a CA-free environment [8].

Here we investigate US-“alone”-related physical stimuli of H9c2 cells. We expose suspended cells to gentle, moderate and extreme US amplitudes. Extreme amplitudes also initiate an increase in transducer temperature; therefore we also investigated the effect of US-related temperature increase on cell viability. Cell viability was also measured following infusion into the micro-device at varying flow regimes in order to optimise the flow rate. Of particular interest to us is the effect of frequency sweeping on cells as a means of controllably stressing cells and potentially increasing membrane permeability.

## Results

Cells were subjected to a variety of US-related stimuli (summarised in Table 1) in order to assess the effect of US alone on cell viability in the absence of CA.

**Table 1:** Summary of the experiments performed to investigate the effect of US-related stimuli on H9c2 cell viability

**Experiment**	**Operating conditions**	**Figure**
Flow rate through the micro-capillary	Inlet flow rate: 1.3–13.0 mL/h	Figure 3
US-induced thermal variations	PZT temperature measurements and CFD simulations of fluid temperature distribution	Figure 4a
	Controls (correspondent PZT temperatures, absence of US)	Figure 4b
Amplitude variations	Driving voltage: 6–29 *V*_*pp*_	Figure 5
Sweep period variations	Sweep period: 0.02–0.50 sec	Figure 1

**Figure 1:**
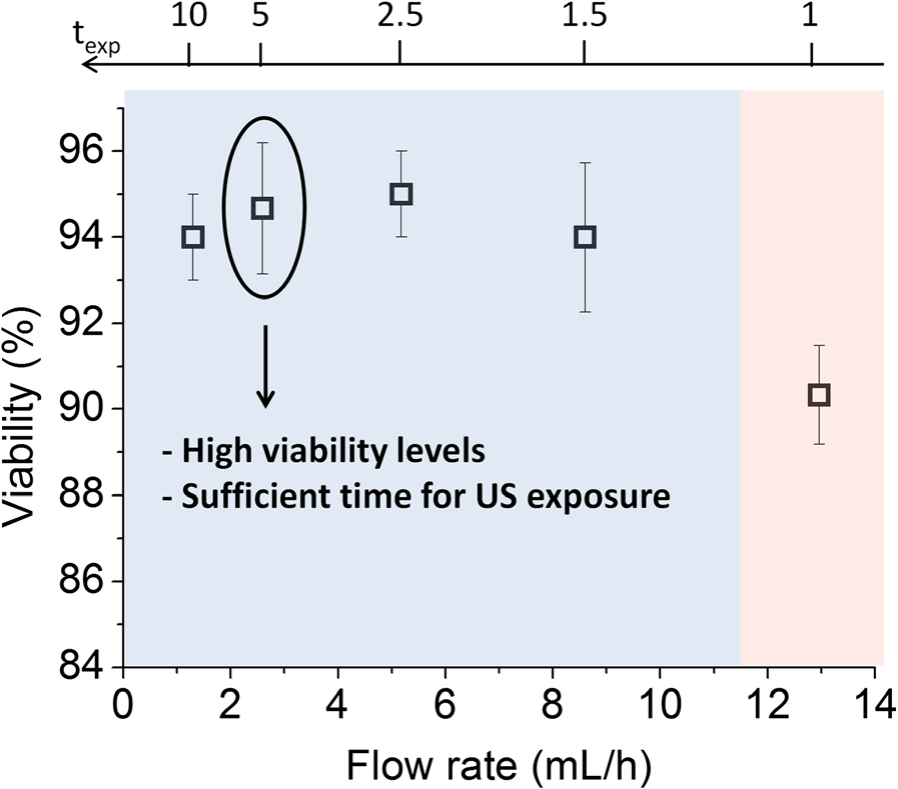
**Effect of fluid flow rate on cell viability.** Cell viability was measured at flow rates ranging from 1.3-13.0 mL/h. At 2.6 mL/h cell viability was uncompromised (control viability = 97±1%), cells were allowed sufficient US exposure (t_exp_ ≅ 5 sec), and trapping was not evident (n = 3).

At 2.6 mL/h viability was uncompromised, likely due to low mechanical stress (Figure 1). This flow rate also provided economical sample collection and processing times whilst allowing sufficient exposure of cells to US (t_exp ≅_ 5 sec) for generating observable bioffects. Furthermore, no cell trapping was evident, providing homogeneous exposure of cells to US.

**Figure 2:**
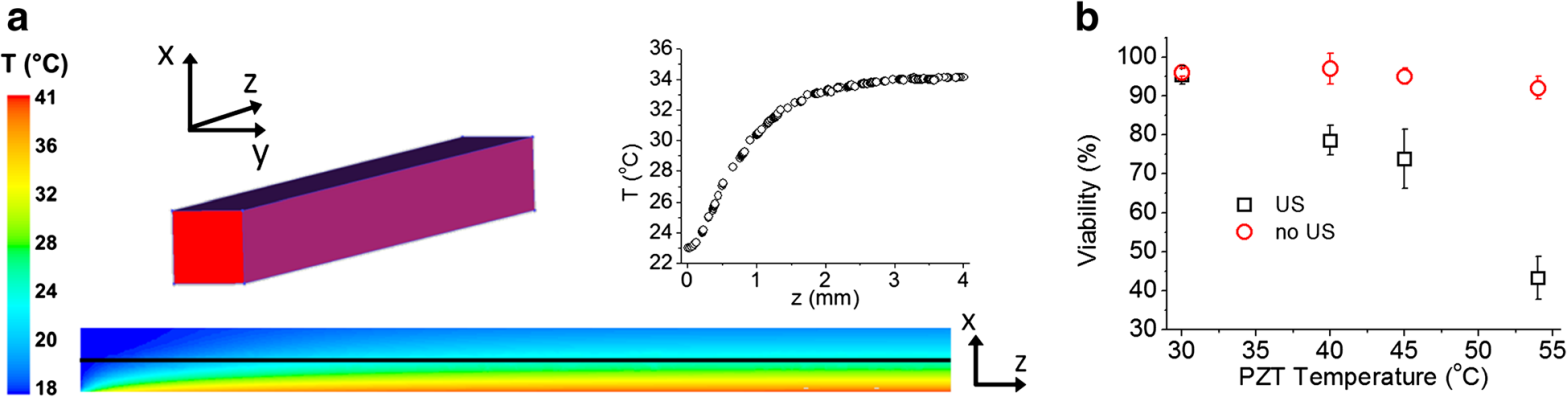
**Effect of temperature on cell viability. a**] Fluid temperature profile along the glass microchannel centerline and contours of fluid temperature determined computationally. **b**] Cell viability during US exposure (black squares), and absence of US (red circle) with PZT substituted by a hot plate at corresponding temperatures (n = 4).

During US applied at the maximum experimental voltage, 29 *V*_*pp*_, the transducer temperature was noted to increase from ambient 23°C to a biologically unfavourable 54°C. However, CFD simulations revealed that the temperature of the liquid medium at the capillary centerline only increased up to a maximum of ~34°C (Figure 2a). To validate the simulations, control experiments (absence of US), replacing the transducer with a hot plate fixed at 54°C, revealed that cell viability was minimally affected (92.12±2.94%), while during US exposure at 29 *V*_*pp*,_ viability reduced to 43.28±5.54% (Figure 2b). This suggests that PZT heating *per se* did not compromise cell viability. However, the CFD simulations did not take into account the effect of acoustic streaming on heat transfer within the fluidic domain, which may have contributed to the reduction in cell viability.

**Figure 3:**
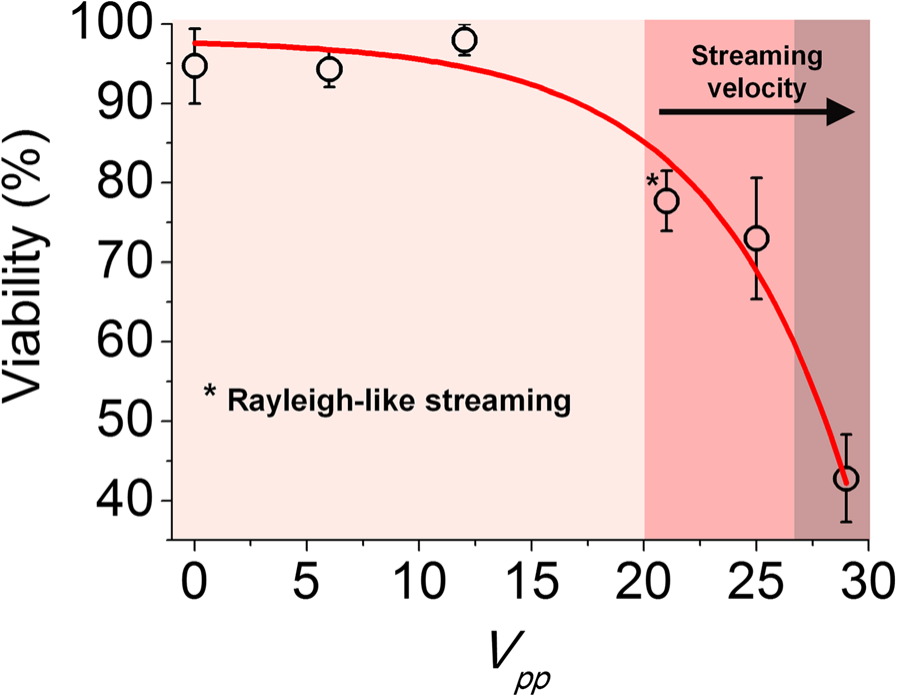
**Effect of US amplitude on cell viability.** Up to ~15 *V*_*pp*_ cell viability was unaffected, a ~17% reduction in viability was measured at 21 *V*_*pp*_ (corresponding to the onset of Rayleigh-like streaming) and a ~60% reduction was measured at 29 *V*_*pp*_ (corresponding to increased streaming velocities) (n = 4).

Cell viability was measured immediately following exposure to US amplitudes ranging from 0–29 *V*_*pp*_, using a fixed frequency sweep period of 0.05 sec. Figure 3 demonstrates that cell viability was not compromised up to ~15 *V*_*pp*_. However a ~17% reduction in cell viability was measured at 21 *V*_*pp*_, corresponding to the onset of streaming, assigned as Rayleigh-like (observed with fluorescent tracers under static conditions), characterised by the formation of toroidal axially centred vortices [10]. A ~60% viability reduction was measured at 29 *V*_*pp*_, corresponding to an observed increase of the streaming velocity. Furthermore, the acoustic pressure (measured by drop-voltage analysis at a fixed *f* = 2.18 MHz) was ~2.05×10^4^×*V*_*pp*_ Pa. This corresponded to pressures in the range 0.12–0.59 MPa, thus suggesting that cavitation may have occurred at *V*_*pp*_ > 20. Notably, Apfel and Holland determined a minimum pressure threshold of ~0.4 MPa for cavitation to occur in water, at a frequency of ~2 MHz [6,11].

**Figure 4:**
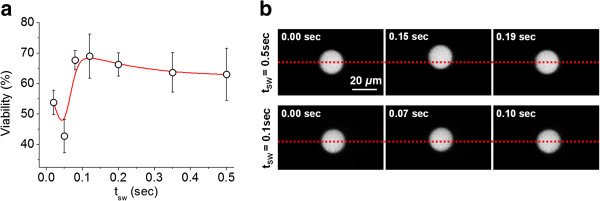
**Effect of frequency sweep on cell viability and bead oscillatory dynamics. a**] Cell viability was unaffected by t_sw_ ≥ 0.08 sec but decrease ≥ 0.08 sec using a frequency range: 2.13-2.40 MHz and at a fixed 29 *V*_*pp*_. **b**] Oscillatory dynamics of 20 *μ*m diameter fluorescent polystyrene beads at a sweep period of 0.5 and 0.1 sec.

At a fixed amplitude (29 *V*_*pp*_) but varying the sweep period (t_sw_ = 0.02-0.50 sec), cell viability was virtually unaffected by frequency sweep periods t_sw_ ≥ 0.08 sec. However, at sweep periods t_sw_ < 0.08 sec cell viability decreased, with minimum viability (~41%) measured at t_sw_ = 0.05 sec (Figure 4a). Under identical acoustic conditions, 20 *μ*m diameter fluorescent beads were observed to rapidly oscillate across a relatively smaller distance away from the nodal plane at t_sw_ = 0.1 sec, compared with t_sw_ = 0.5 sec where bead oscillation was visibly slower over increased distances away from the nodal plane (Figure 4b).

## Discussion

The effect of individual US-related physical parameters (fluid flow rate, US heat generation, amplitude and frequency sweep period) on H9c2 cell viability was assessed within a microfluidic device. The optimised flow rate did not inflict any detectable mechanical stress, and thus high cell viability was maintained. Moreover cells were allowed sufficient exposure to US in order to elicit bioeffects, whilst providing economical sample processing times and minimising cell trapping. High cell viability was maintained at amplitudes where streaming was not evident. However, when more extreme amplitudes were employed, streaming velocities increased and cell viability significantly decreased. Extreme amplitudes also initiated an increase in PZT temperature, however cell viability was unaffected by this increase due to heat dissipation, confirmed by controls and CFD simulations. Longer duration frequency sweeps were identified to have little or no effect on cell viability, whereas short sweeps resulted in reduced cell viability. This effect may be attributed to mechanical stress generated by rapid oscillatory movements of the cell within the fluidic domain [12]. Notably, experiments with fluorescent tracer beads revealed that bead oscillation frequency increased with reducing the sweep interval, which may explain the reduction in cell viability at the shorter t_sw_. However, an in depth investigation into the effects of frequency sweeping on cell viability is currently underway in our laboratories.

## Conclusion

Our CA-free investigation into the effects of US on cell viability has enabled us to discriminate between US-related stimuli that do not compromise cell viability and stimuli that significantly reduce cell viability within our micro-device. Our findings may be of potential interest to researchers in the field of US-induced intracellular drug delivery and ultrasonic manipulation of biological cells.

## Methods

**Figure 5:**
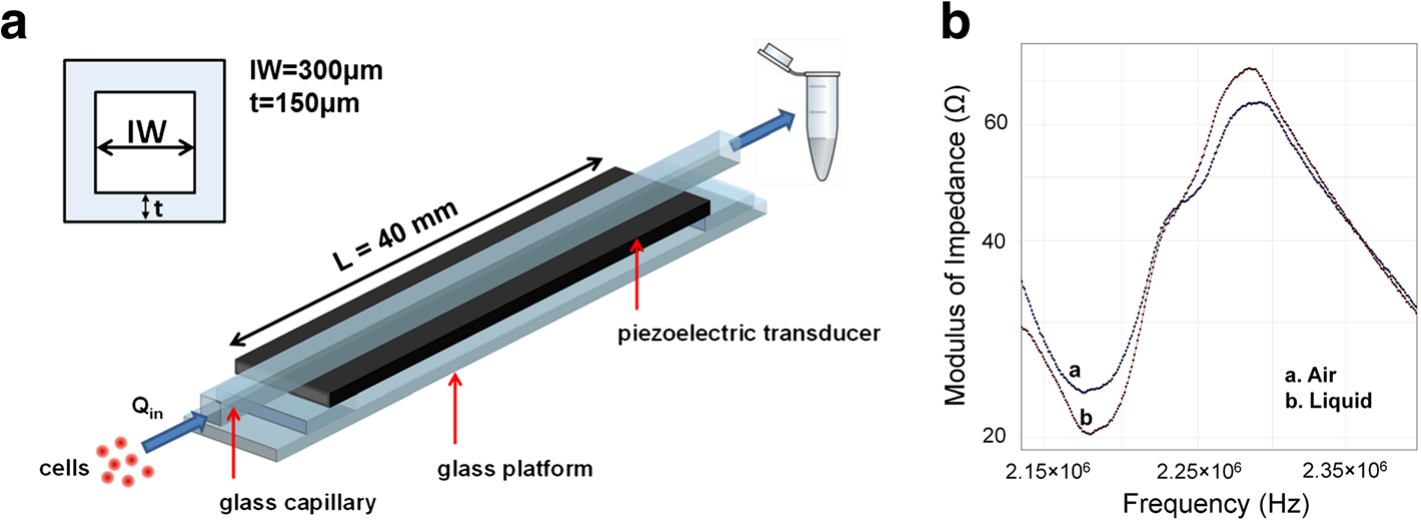
**Microfluidic device. a**] device comprising of a squared cross-section glass capillary (length: 50 mm, internal width: 300 *μ*m and wall thickness: 150 *μ*m), coupled to a PZT transducer (length: 40 mm, width: 9 mm and thickness: 1 mm) and mounted on a glass platform. **b**] Modulus of impedance (Ω) for the air-filled and the liquid-filled micro-capillary, respectively. Frequency range: 2.13-2.40 MHz.

The micro-device (Figure 5a), comprising of a squared cross-section borosilicate glass micro-capillary (length: 50 mm, internal width: 300 *μ*m, wall thickness: 150 *μ*m; VitroCom, Ilkley, UK), was acoustically coupled to a piezoelectric transducer (PZT; PZ26 Ferroperm, Kvistgard, Denmark) using glycerol. The transducer (length: 40 mm, width: 9 mm and thickness: 1 mm) was fixed to a glass platform and driven by an RF power amplifier (240 L ENI, New York, USA) fed from a signal generator (TG103 TTI, Cambridgeshire, UK). A time varying ultrasonic field was generated within the capillary and the operating frequency determined through electrical impedance measurements (C-60 impedance analyser, Cypher Instruments Ltd., London, UK) of the capillary both air-filled and fluid-filled (Figure 2b).

H9c2 cardiac myoblasts were grown in Dulbecco’s Modified Eagle Medium (DMEM) culture medium supplemented with 10% (v/v) foetal calf serum and 1% (v/v) penicillin-streptomycin (media and supplements purchased from Fisher Scientific, Loughborough, UK). Cells were maintained at 37°C, 5% CO_2_ in air with 95% humidity. Cells were routinely harvested and suspended at a density of 2×10^6^ cells/mL in serum free DMEM within a 1 mL sterile, plastic syringe (BD Bioscience, Oxford, UK). Cells were infused into the device using a syringe pump (KD100, KD Scientific Inc., Holliston, USA) and subjected to ultrasound-related physical stimuli. Cells were captured in 1 mL sterile tubes, followed by counting and viability assessment using a Neubauer haemocytometer (depth: 0.1 mm, area: 0.04 mm^2^) and trypan blue exclusion dye. All viability measurements were in triplicate or greater.

To optimise the flow rate, cell viability was measured following infusion into the device at a range of flow rates (1.3-13.0 mL/h), which were prior calculated in order to: i) provide sufficient exposure of cells to US, ii) provide economical cell collection and processing times, iii) minimise flow-induced mechanical stress on cells and iv) minimise cell trapping.

To assess US-related thermal effects on cell viability, cells were infused into the device at a fixed flow rate (2.6 mL/h) and exposed to US (6–29 *V*_*pp*_), whilst thermocouples were attached to the transducer, and temperatures recorded using a thermometer (HH11 Omega®, Manchester, UK). Controls were produced in the absence of US by replacing the transducer with a hot plate (Fisher Scientific, Loughborough, UK) at identical temperatures to the recorded transducer temperatures. Additionally, computational fluid dynamic (CFD) simulations were performed to predict the transfer of heat from the transducer to the cell medium within the capillary.

The effect of US amplitude on cell viability was investigated by varying the *V*_*pp*_, ranging from 0–29 *V*_*pp*_, using a fixed frequency sweep period of 0.05 sec in the frequency range 2.13-2.40 MHz. Additionally, flow visualisation experiments, using 1 *μ*m diameter fluorescent tracers (Polysciences, Inc., Warrington, USA), were performed to characterise the fluid dynamic environment under “gentle” (6 *V*_*pp*_) to “extreme” (29 *V*_*pp*_) US amplitudes. The acoustic pressure within the capillary was measured through drop-voltage analysis [9], using 20 *μ*m diameter fluorescent polystyrene beads. A fixed resonance frequency of 2.18 MHz was set in this case, due to the difficulty in obtaining acoustic pressure values during frequency sweeping.

The effect of frequency sweep duration on cell viability was investigated by varying the sweep period (0.02-0.50 sec) at a fixed voltage (29 *V*_*pp*_).

## Competing interests

The authors declare that they have no competing interests.

## Authors’ contributions

Conceived, designed and performed experiments: DA and DC. Data analysis: DA and DC. Wrote the paper: DA and DC. Device design: PGJ. Invaluable advice provided on microfluidics: XZ, acoustics: JL and MH and biology: PAT. All authors read and approved the final manuscript.
